# Locus of Enterocyte Effacement: A Pathogenicity Island Involved in the Virulence of Enteropathogenic and Enterohemorragic *Escherichia coli* Subjected to a Complex Network of Gene Regulation

**DOI:** 10.1155/2015/534738

**Published:** 2015-02-02

**Authors:** Fernanda M. Franzin, Marcelo P. Sircili

**Affiliations:** ^1^State University of Campinas, Campinas, SP, Brazil; ^2^Laboratory of Genetics, Butantan Institute, Vital Brazil Avenue, 05503-000 Sao Paulo, SP, Brazil

## Abstract

The locus of enterocyte effacement (LEE) is a 35.6 kb pathogenicity island inserted in the genome of some bacteria such as enteropathogenic *Escherichia coli*, enterohemorrhagic *E.coli*, *Citrobacter rodentium*, and *Escherichia albertii*. LEE comprises the genes responsible for causing attaching and effacing lesions, a characteristic lesion that involves intimate adherence of bacteria to enterocytes, a signaling cascade leading to brush border and microvilli destruction, and loss of ions, causing severe diarrhea. It is composed of 41 open reading frames and five major operons encoding a type three system apparatus, secreted proteins, an adhesin, called intimin, and its receptor called translocated intimin receptor (Tir). LEE is subjected to various levels of regulation, including transcriptional and posttranscriptional regulators located both inside and outside of the pathogenicity island. Several molecules were described being related to feedback inhibition, transcriptional activation, and transcriptional repression. These molecules are involved in a complex network of regulation, including mechanisms such as quorum sensing and temporal control of LEE genes transcription and translation. In this mini review we have detailed the complex network that regulates transcription and expression of genes involved in this kind of lesion.

## 1. Introduction

Attaching and effacing (A/E) is a histological lesion that occurs in intestinal epithelial cells as consequence of infection by a group of pathogens that includes enteropathogenic* Escherichia coli* (EPEC), enterohemorrhagic* Escherichia coli* (EHEC), rabbit diarrheagenic* Escherichia coli* (RDEC), the murine pathogen* Citrobacter rodentium, *and* Escherichia albertii *[1]. The A/E lesion was first described by Moon et al. 1983 [2] and is characterized by intimate attachment of bacteria to the enterocyte membrane, destruction of the brush border microvilli, and striking cytoskeletal changes that include accumulation of polymerized actin beneath the site of attachment, forming pedestal-like structures [3–6]. The intimate attachment of A/E pathogens to enterocyte surface is mediated through interactions of the bacterial outer membrane protein intimin and the translocated intimin receptor (Tir) [7]. Tir and other effector proteins are delivered to the host cell via a type III secretion system (T3SS).

The genes responsible for A/E lesion phenotype are encoded on a 35.6 Kb pathogenicity island known as locus of enterocyte effacement (LEE) [2, 6, 8, 9], which comprises the structural components of type III secretion system (TTSS), regulators, chaperones, and effectors secreted by the TTSS. LEE was first described in EPEC E2348/69 by Mcdaniel et al. 1995 [10] and consists of 41 ORFs functionally organized into five operons called* LEE1*,* LEE2*,* LEE3*,* LEE4*, and* LEE5* [11].* LEE1*,* LEE2*, and* LEE3* harbors the* esc* and* sep* genes, encoding the major components of type III secretion system [12].* LEE1* harbors the LEE encoded regulator (*ler*) gene, which encodes Ler, a DNA binding protein which acts as a main transcriptional regulator of the LEE region, modulating the expression of other* LEE* operons [13]. The* LEE5* operon contains the EPEC attaching and effacing (*eae*) and translocated intimin receptor (*tir*) genes, which encodes the adhesion intimin and its receptor Tir, respectively. The effector proteins translocated via type III secretion system to the host cell are encoded by* espAespB, espD, sepL, espF, *and* escF* genes, located at* LEE4* operon [9, 14, 15].

Enteropathogenic* Escherichia coli* (EPEC) are among the most important pathogens causing persistent diarrhea in children and adults worldwide [6, 16–18]. Outbreaks of EPEC were frequent in developed countries in the 1940s and 1950s [19] but have largely disappeared from industrialized countries. However, EPEC continues to be an important cause of child mortality in developing countries. In Brazil and other developing countries, EPEC is associated with at least 20% of the cases of diarrhea [18, 20–22]. EPEC have been divided into two subgroups: typical EPEC (tEPEC) and atypical EPEC (aEPEC), based on the presence or absence of the EPEC adherence factor plasmid (pEAF), respectively [6, 23]. Atypical EPEC has been shown to be more prevalent than typical EPEC in developed and developing countries and is considered an emergent cause of endemic diarrhea in children and diarrhea outbreaks in adults [3–5].

Enterohemorrhagic* Escherichia coli* (EHEC) are a leading cause of bloody diarrhea and hemolytic uremic syndrome (HUS) throughout the world [24]. EHEC strains of the O157:H7 serotype are the most important EHEC pathogens in North America, the United Kingdom, and Japan [6]. This strain has been associated with numerous outbreaks worldwide and constitutes a serious public health threat. EHEC produces a potent Shiga toxin (Stx) that is responsible for the major symptoms of hemorrhagic colitis and HUS [25].

Although their sites of infection are different, EPEC infects the small bowel and EHEC colonizes the large bowel, these two* E. coli *pathotypes share a similar mode of infection, since both are A/E pathogens [6]. EPEC and EHEC must control virulence gene expression during infection aiming for maximal energy efficiency. Regulation of LEE gene expression is a highly complex and coordinated process, dependent on environmental conditions, quorum sensing, and several regulators and regulatory pathways, in a direct or indirect way.

This review is focused on summarizing the current knowledge of regulation of LEE gene expression in EPEC and EHEC and the molecules involved in this regulation are listed in Table 1.

## 2. LEE-Encoded Regulators

The central regulator which controls the expression of LEE genes is Ler, a 15 kDa protein encoded by the first gene of the* LEE1* operon, which is a HNS-like regulatory protein [11, 13, 26–29]. Ler is essential for the formation of A/E lesions, since nonpolar* ler* mutants of EPEC and EHEC were unable to form A/E lesions on HEp-2 cells [13]. In addition, these* ler* mutants failed to express type III secreted proteins, which means that all genes known to be important in A/E lesion formation are regulated by Ler [13]. Since Ler expression is central to regulation of virulence genes, it is true that the regulation of Ler expression is important to pathogenesis. In fact, Ler expression is under control of numerous regulatory factors, encoded in or out of the LEE region.

Global regulator of LEE activator (GrlA) and global regulator of LEE repressor (GlrR), two other LEE-encoded regulators, were described by Deng et al. 2004 [30] in a* C. rodentium* background. These regulators are encoded by the* grlRA* operon located between the* rorf3* gene and the LEE2 operon in LEE. GrlA has been shown to play a key role in the regulation of* ler* and thus in the expression of LEE genes. The observation that transcription of* LEE1*,* LEE2*, and* LEE5* was diminished in* grlA* mutants and the transcription of* LEE1* was mildly elevated in* grlR* mutants has suggested the activator and repressor roles of LEE gene transcription to GrlA and GrlR, respectively. GrlA has been shown to bind on* LEE1* promoter and positively regulate Ler expression. Ler also controls the expression of the* grlRA* operon, forming a positive regulatory loop that ensures the appropriate level of Ler to activate LEE gene expression [31]. Using a yeast two-hybrid system, it was reported that GrlR specific binds to GrlA [32]. This interaction represses LEE gene expression by preventing GrlA interaction with* LEE1* promoter [33] or rather prevents repression by GrlR, which inhibits LEE gene transcription specifically and independently of its interaction with GrlA [34].

## 3. Non-LEE-Encoded Regulators

As the LEE is an essential virulence determinant of pathogenesis of EPEC and EHEC, the expression of its genes is under control of a myriad of transcriptional regulators.

In typical EPEC, the LEE genes are regulated by a region located on a 90 kb EPEC adherence factor plasmid (EAF plasmid) called plasmid encoded regulator (Per). The* per* locus includes* perA*,* perB,* and* perC* transcribed as a single polycistronic message [35]. Mellies et al. (1999) demonstrated that Per indirectly activates* LEE2* and* LEE3* in this pathogen. PerC directly activates* LEE1* promoter and then Ler acts on* LEE2*,* LEE3*,* LEE4*, and* LEE5* promoters. Bustamante et al. (2001) have shown that* perC *gene is indirectly required for* LEE2 *and* LEE3* transcription. PerC have been reported to induce* ler* transcription independently of GrlA [36]. In EHEC, a family of functional prophage-encoded PerC homologous proteins, called PchA, PchB, and PchC, plays an important role in* ler *expression [37]. Moreover, a PerC-like protein family identified from EHEC genome analyses, PerC1 (also termed PchABC), can also activate both promoters in a manner similar to that of EPEC PerC. PerC1 proteins are able to mediate activation of the EPEC and EHEC* LEE1 *promoters in a PerC-like manner, while PerC2 (and PerC3) seems to be inactive. This finding correlates with the fact that PerC1 is more closely related to PerC than is PerC2, and much more so than PerC3 [38].

H-NS is a 15.6 kDa nucleoid-associated protein that binds nonspecifically to DNA and modulates, mostly negatively, the expression of genes involved in many different cellular processes, including virulence [34]. H-NS plays an important role in the repression of LEE genes [13, 28, 30]. It binds to EPEC* LEE1*,* LEE2*,* LEE3*, and* LEE5* regulatory regions, directly controlling LEE transcription [29, 39]. It has been shown that different transcriptional regulators can counteract the repression mediated by H-NS on the expression of virulence genes [40–42]. Ler can neutralize the silencing exerted by H-NS on LEE promoters [28, 30, 36] but not on its own promoter that is also repressed by H-NS [30]. Ler binds with higher affinity than H-NS to the same region of* LEE5* and* LEE2*, disrupting H-NS-containing nucleoprotein complexes [27, 28, 43]. The* E. coli* stringent starvation protein A (SspA) is involved in downregulation of the cellular H-NS level during stationary phase [36, 44]. It also has a role in positive regulation of virulence genes in EHEC, since it was demonstrated that in the absence of SSpA, EHEC produces high levels of H-NS which represses the expression of LEE genes, rendering cells incapable of forming A/E lesions [45].

Friedberg et al. (1999) demonstrated that integration host factor (IHF) is essential for expression of LEE-encoded type III secretion genes and directly activates LEE1 transcriptional unit. IHF acts by disrupting the Ler repression by HNS binding specifically upstream from the* ler* promoter to activate expression of* ler*, orf3, orf5, and orf2 directly [26]. The overcoming of H-NS silencing of the* ler* promoter requires the combined antirepressor activity of IHF with PerC or GrlA [34].

Another nucleoid-associated protein that has an impact upon virulence gene expression in EPEC is the factor for inversion stimulation (Fis). Fis was reported to positively regulate* ler *and* LEE4* expression. Goldberg et al. (2001) [45] have shown that major EPEC secreted proteins (EspA, EspB, and EspD) were found to be significantly reduced and that* ler* expression was abolished in the Δ*fis* mutant, resulting in inability to induce A/E lesion. However,* tir *and intimin expressions were found to be unaffected in these mutants, which suggest that the promoter of this operon is not regulated by Fis.

In EPEC, the ribosome-binding GTPase BipA has been shown to modulate virulence expression by increasing LEE transcription, since several key proteins encoded by LEE had lower levels in the absence of BipA. Transcription of* ler* was reduced in the absence of BipA, suggesting that this GTPase acts most likely indirectly through activating expression of Ler [46].

Recently, Flockhart et al. (2012) [47] have reported a new LEE regulator, termed RgdR, that is encoded in CP-933 prophage of O157:H7 and activates* LEE1* transcription in the presence or absence of Ler. Sharma and Zuerner (2004) [48] have reported that EHEC O157:H7 mutants of* hha* gene showed 10-fold increase of* ler *transcription, suggesting that Hha acts as a repressor of* ler* transcription by binding to its promoter.

Alternative sigma factor 54 (RpoN) is one of seven RNA polymerase sigma subunits in* E. coli* required for promoter-initiated transcription. RpoN was shown to positively regulate the expression of genes in all five LEE operons [49], perhaps through indirect activation of* grlA*. RpoN may also regulate LEE through GadE, since* gadE* expression was upregulated in Δ*rpoN* mutant [48]. GadE is a transcriptional activator of the glutamate decarboxylase (GAD) system of* E. coli* and has been reported to repress LEE expression in a* ler* dependent fashion [50].

The Cpx two-component system regulates an envelope stress response adapted by* Escherichia coli *to monitor and maintain cell envelope integrity. This pathway also has a direct effect on transcription of virulence genes and virulence regulators in different pathogens. Activation of the Cpx pathway negatively affects EPEC TTSS by downregulating the expression of components of the translocation apparatus as well as the receptor Tir [51]. Cpx pathway is capable of inhibiting LEE expression in a nonpathogenic* E. coli* background, which suggests that Cpx-mediated inhibition of LEE gene expression and TTSS is independent of Ler. Cpx response might also inhibit T3SS in EPEC at posttranscriptional levels and DegP is a contributing factor in this inhibition [52].

Whereas our knowledge about the mechanisms of transcriptional regulation of the LEE is vast, our understanding of posttranscriptional and posttranslational regulation is limited. In term of posttranscriptional and posttranslational regulation, RNase E generates the sepL and espADB transcripts by splicing at the C-terminal end of sepL in the precursor sepLespADB transcript of EHEC [53]. CsrA, an RNA-binding protein [54], is a posttranscriptional regulator molecule in A/E pathogens that activates and represses the expression of LEE-encoded genes in a concentration-dependent manner [55]. CsrA exerts its effect by binding to the leader segment of the* sepLespADB *mRNA and enhancing the steady-state transcript and protein levels.

ClpX and ClpP were suggested to be positive regulatory genes that control expression of LEE in EHEC O157. It was shown that O157:H7 Sakai clpPX mutant strongly impaired the secretion of virulence proteins by TTSS and repressed transcription from all the LEE promoters. This positive regulation partially depends on the intracellular levels of RpoS, a substrate for the ClpXP protease, since the Δ*rpoS* mutation in O157:H7 Sakai enhanced the transcription from all the LEE promoters and the secretion of virulence proteins, and it could partially suppress the defects of the Δ*clpPX* mutation. However, the regulation of LEE by ClpXP also occurs in a sigma-(S-) independent pathway that involves the negative regulation of GrlR levels in EHEC [37, 56].

The stress sigma factor RpoS has also been involved in regulatory cascades controlling LEE expression [37, 57]. The effects of RpoS on expression of the LEE virulence genes appear to vary depending on strain backgrounds and experimental conditions. Laaberki et al. (2006) [58] have reported that the* LEE3 *operon and* tir* are positively regulated by RpoS in EHEC strain 86-24. However, in EHEC O157:H7 Sakai strain, LEE expression is enhanced in* rpoS* mutants [37, 56, 58] due to increases in* ler *mRNAs levels [58], indicating that RpoS could repress* ler *expression. RpoS acts by binding to* ler *promoter to activate the transcription through a pathway different than that used by H-NS. In turn, RpoS translation depends on the high levels of DsrA, a noncoding RNA (ncRNA) which modulates the expression of RpoS and H-NS.

Noncoding RNAs (ncRNAs) have recently emerged as important regulators involved in controlling the expression of regulatory and effector genes [59]. DsrA acts by an antisense base-pairing mechanism on H-NS mRNA, decreasing its stability, and on RpoS mRNA, stimulating its translation [60, 61]. DsrA enhances* ler* expression by affecting the levels of functional transcripts and the DsrA-mediated activation of LEE requires Ler. DsrA effects on the expression of intimin and EspA would mainly be due to its effect on* ler* expression and the DsrA activation of* ler* expression requires RpoS [58]. DsrA also affects the H-NS levels; however RpoS- and HNS-dependent pathways separately regulate* ler *expression.

Hfq is a conserved RNA-binding protein that regulates diverse cellular processes through posttranscriptional control of gene expression, often by functioning as a chaperone for regulatory sRNAs in many bacterial species [62]. Hfq has been described as a negative regulator of the expression of all of the 41 LEE genes acting directly upon* ler* mRNA, as translation of this transcript is increased in the absence of Hfq in EHEC EDL933 [63]. Hansen and Kaper (2009) have shown that Hfq negatively affects LEE expression in exponential phase by regulating the expression of the LEE-encoded regulators GrlA and GrlR at the posttranscriptional level by destabilizing* grlRA* mRNA. Hfq negatively affects LEE expression in stationary phase independent of GrlA and GrlR in EHEC EDL933 [64]. However, Kendall et al. (2011) have reported that Hfq plays a regulatory role in EHEC 86-24 that is different from what has been reported for EHEC strain EDL933, acting as a positive regulator of the TTSS expression and A/E lesion formation in this strain [65]. Taken together, these studies point to the possibility that this kind of regulation might be different even in closely related strains.

Recently, Gruber and Sperandio noticed that LEE and espFu genes are posttranscriptionally regulated by the GlmY/GlmZ sRNAs. GlmY and GlmZ promote cleavage of the intergenic region between* espJ* and* espFu* to allow the translation of EspFu [66].

## 4. Environmental Signals Such as Carbon Sources

There are recent studies correlating some carbon sources to LEE regulation. Takao et al. 2014 noticed that LeuO can recognize butyrate and activate a signaling cascade. LeuO expression induces prolonged activation of the promoter of LEE1 operon, including the ler gene, as well as virulence mechanisms such as microcolony formation. Activation of the LEE1 promoter by LeuO depends on another regulator, called Pch. The response of the leuO promoter to butyrate requires two virulence regulators, Pch and Ler, in addition to Lrp. Pch, Ler, and Lrp bind the upstream region of the leuO promoter. Thus, leuO is involved in butyrate-enhanced expression of LEE genes through a positive feedback mechanism, but its expression and action on the LEE1 promoter are dependent on the virulence regulators Pch and Ler [67].

Another sugar that is highly abundant in the intestine is fucose. Recently, Pacheco et al. described a mechanism of fucose recognition and virulence regulation. They described one novel two-component signal transduction system, named FusKR, where FusK is the histidine sensor kinase and FusR the response regulator. FusK senses fucose and controls expression of virulence and metabolic genes. This fucose-sensing system is required for robust EHEC colonization of the mammalian intestine [68]. Ethanolamine can act as a signal to modulate virulence. The EutR transcription factor, known to be the receptor of EA, is only partially responsible for this regulation, suggesting that yet another EA receptor exists. Ethanolamine (EA) is present in the large intestine due to the turnover of intestinal cells. Kendall et al. 2012 showed that during growth at early log phase,* ler* expression in WT EHEC was increased over 10-fold when EA was added to the medium. At mid- and late log phases, the addition of EA did not significantly affect the level of* ler* transcription [69]. Njoroge et al., 2013 studied the role of Cra and KdpE and concluded that although these regulators clearly have individual targets, they also share either convergent or divergent regulation of subsets of genes. Cra positively regulate the genes encoding gluconeogenic enzymes, and negatively regulate genes encoding glycolytic enzymes. Cra and KdpE converge in the regulation of the LEE T3SS and several non-LEE-encoded effectors, which agrees with the fact that the expression of these genes should be coupled, even though they are found in different chromosomal regions. Both transcription factors activate* LEE1* expression under gluconeogenic conditions. Another factor involved in the trigger of AE lesion is the phosphorylation of Tir and the accessory protein EspFu/Tccp [70].

## 5. Quorum Sensing and Signaling Systems

Several signaling systems have been well studied between host-cell pathogens interactions; among them there are at least four involved in chemical signaling described in the literature: (1) AI-1 System, (2) AI-2 System, (3) AI-3/epinephrine system, and (4) indole. At least two systems were described in mechanisms involved in LEE gene regulation in EPEC and EHEC strains.

The first study reporting quorum sensing regulation of LEE in EHEC and EPEC was performed by Sperandio et al. (1999). In this pioneering study, they noticed that the transcription of* LEE1* and* LEE2* was increased in the presence of preconditioning media, suggesting that EPEC and EHEC secrete AI-2 [57]. At that time point it was proposed that AI-2 acts by activating these operons and thus positively regulating the expression of TTSS genes. Another study performed by Sircili et al. 2004 using one E2348/69 EPEC strain noticed that* qseA* and* luxS* were involved in LEE genes transcription altering TTSS expression and reducing A/E lesion formation [71]. Later studies noticed that AI-2 was not involved in this mechanism of regulation, and the molecule involved was actually AI-3.

## 6. AI-1 System and Indole

*Escherichia coli* does not harbor one* luxI* homologue and does not produce the autoinducer, although it harbors one* luxR *homologue which is called* sdiA. *SdiA is responsible for sensing the environmental AI-1 signals produced by other microorganisms [72]. Studies with SdiA mutants have shown that this regulator may be involved in biofilm formation in* E. coli*. SdiA has been proposed to interact with indole [73], in addition to acyl homoserine lactones (AHLs) from other bacteria. However, conclusive data demonstrating that SdiA senses that indole is lacking. In* E. coli,* SdiA, named after its ability to suppress cell division inhibitors, is a 240-amino-acid protein that belongs to the LuxR family of transcriptional regulators. Sheng et al. 2013 noticed that deletion of* sdiA* removes the ability to sense AHLs and regulate LEE and* gad* gene expression, and as a result the* sdiA* mutant did not grow well in rumen and was defective, compared to the WT, for colonization of the RAJ mucosa of these cattle. They report that these animals, still harbor AHLs in their rumen, and these AHLs can still repress* ler* and increase* gad* gene expression in an SdiA-dependent way [74]. One recent work of Bommarius et al., showed that indole derivatives also represses production of LEE virulence factors and inhibit pedestal formation on mammalian cells [75].

## 7. AI-3/Epinephrine/Norepinephrine System

AI-3/epinephrine/norepinephrine system was first described in enterohemorrhagic* E. coli* O157:H7 (EHEC) strains [76]. It was initially thought that the gene responsible for production of the molecule was* luxS*, since* luxS* mutant has diminished AI-3 production. Another study performed by Sperandio's group showed that* luxS* mutants have metabolic deficiencies, and* luxS* is not responsible for AI-3 production [77]. The AI-3 signal is an aromatic compound, and the host hormones epinephrine and norepinephrine can restore AI-3-dependent phenotypes in EHEC. Based on that, it has been proposed that this system is involved in host cell-bacteria communication, and interkingdom signaling. The AI-3 receptor, QseC sensor kinase [78] has been extensively described in EHEC and other species. Subsequent studies showed that AI-3/epinephrine/norepinephrine system is not restricted to* E. coli *strains [79].

EHEC and EPEC* luxS *mutant both present diminished AI-3 production and do not express the LEE-encoded T3SS system at normal levels, nonetheless still form AE lesions on epithelial cells that were indistinguishable from those seen with wild type.* luxS *mutants were still responding to eukaryotic cell signals to activate expression of the LEE genes. These signals were identified as the hormones epinephrine and norepinephrine. Epinephrine and norepinephrine can substitute for AI-3 to activate transcription of the LEE genes, type III secretion, and AE lesions on epithelial cells.

In EHEC, the mammalian hormones epinephrine and norepinephrine, which are released by the host during stress, are sensed by the QseC receptor to regulate bacterial virulence genes [80, 81]. The* qseC *mutant is attenuated for virulence, which underscores the importance of this interkingdom communication to the development of disease [78]. Regarding atypical EPEC, we performed studies using one* qseC* mutant and noticed a similar pattern of regulation (unpublished results).

Concerning the EHEC AI-3/epinephrine/norepinephrine signaling system cascade, there are other transcriptional regulators involved; however the timing and interactions still need more studies to be elucidated. We can cite some of these molecules, such as QS* E. coli *regulator (QseA) [80]. QseA is transcriptionally activated through QS and, in turn, binds to and directly activates transcription of the LEE-encoded regulator Ler. Interestingly,* qseA* mutant in EPEC has a similar reduction in TTSS, but has defect in motility [71], suggesting a different kind of regulation between these two pathotypes, at least regarding these two strains tested. Another two-component system, named QseBC is responsible for the transcriptional activation of the flagella regulon in response to QS. Early studies indicated that an isogenic mutant in the* qseC *sensor kinase was unable to respond to AI-3 or epinephrine given exogenously [78]. There are other genes recently described in this signaling cascade, and it is important to notice the identification of* qseE *and* qseF *(encoding a second two-component system), which are involved in regulating AE [81]. Additionally, QseE has also been shown to sense epinephrine, sulphate, and phosphate, but not AI-3 [82]. The AI-3-dependent QS signaling cascade is present in all Enterobacteriaceae (*E. coli, Salmonella *spp.,* Shigella *spp., and* Yersinia *spp.). The most striking feature is that the genes encoding the transcriptional factors of this cascade are always in the exact same context in the chromosome of all these strains and share high levels of identity among these different species, suggesting that this signaling cascade is functionally conserved in Enterobacteriaceae.

## 8. Concluding Remarks

**Figure 1:**
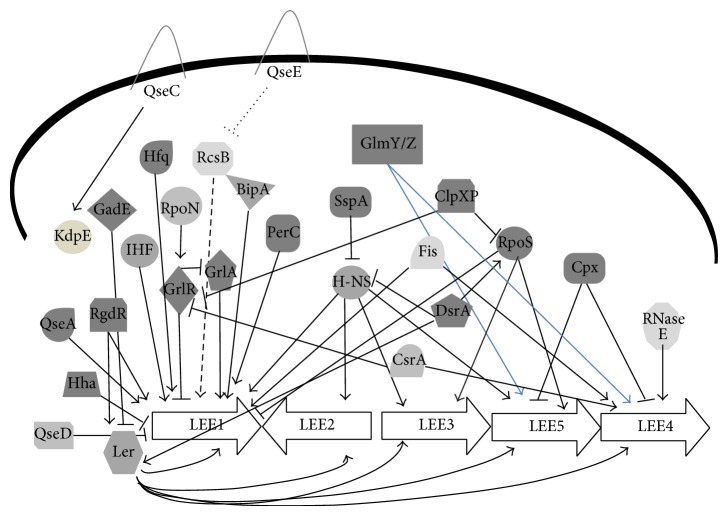
Schematic representation of LEE genes transcription regulation. The pointed arrows indicate activation, and repression is represented by blunt arrows.

Elucidation of the kinetics of AE lesion formation demonstrated that this complex phenotype is tightly regulated in response to environmental signals, and there are several molecules and mechanisms involved in this regulation. It is important to notice that there are a lot of molecules and environmental signals involved in this pathogenicity island regulation, and this very complex cascade of regulators helps to activate or repress virulence, sensing the conditions of the environment and pointing to the colonization process. We must understand these mechanisms and these regulators because these molecules can be targets to block the virulence factors transcription and translation, consecutively preventing the colonization. We demonstrate the complex signaling cascade on a scheme (Figure 1).

## Figures and Tables

**Table 1:** Regulators involved in LEE genes transcription.

Regulator	Bacteria	Regulatory mechanism	Phenotype/response	References
*LEE-encoded *				
GrlA	*C. rodentium*; EPEC, EHEC	Binding *LEE1* promoter	Activates LEE expression through Ler	Deng et al., 2004 [30]
GrlR	*C. rodentium*; EPEC, EHEC	Binding to GrlA preventing it to bind *LEE-1* promoter	Represses Ler/LEE expression	Deng et al., 2004 [30]
Ler	A/E pathogens	Binding to LEE promoters	Activates expression of all LEE operons	Elliott et al., 2000 [13]

*Non-LEE encoded *				
BipA	EPEC, EHEC	Activates *ler* transcription	Activates LEE expression through Ler	Grant et al., 2003 [46]
Cpx	EPEC, EHEC	Downregulates *LEE4* and LEE-5 transcription	Represses LEE expression	Macritchie et al., 2008 [51]
Fis	EPEC	Activates *ler* and *LEE4* transcription	Activates LEE expression	Goldberg et al., 2001 [45]
GadE	EHEC		Represses LEE expression	Vanaja et al., 2009 [50]
Hha	EHEC	Binding *ler* promoter	Represses Ler transcription	Sharma and Zuerner, 2004 [48]
H-NS	EPEC, EHEC	Binding to *LEE1*, *LEE2*, *LEE3* and *LEE5* regulatory region	Represses Ler expression	Bustamante et al., 2001 [28]
IHF	EPEC	Binding upstream to *ler* promoter	Activates *ler*, orf3, orf5, and orf2 expression	Friedberg et al., 1999 [26]
Pch/PerC 1, 2^*^, 3^*^	EHEC	Enhancing *ler* transcription	Activates LEE expression ^*^represses LEE1 transcription	Iyoda and Watanabe, 2004, [44] Porter et al., 2005 [38]
PerC	Typical EPEC	Activating *LEE1* promoter	Activates LEE expression	Mellies et al., 1999 [11]
RgdR	EHEC	Activating *LEE1* transcription	Activates LEE expression	Flockhart et al., 2012 [47]
RpoN	EHEC	Activating *grlA*, repressing *gadE *	Activates LEE expression	Riordan et al., 2010 [49]
SspA	EHEC	Repressing H-NS expression	Activates LEE expression	Goldberg et al., 2001 [45]
FusK/R	EHEC	Represses *LEE1 *	Represses LEE expression	Pacheco et al., 2012 [68]
EutR	EHEC	Activates *LEE1 *	Activates LEE expression	Kendall et al., 2012 [69]
LeuO	EHEC	Activates *LEE1 *	Activates LEE expression	Takao et al., 2014 [67]
SdiA	EHEC	represses *LEE1 *	represses LEE expression	Sheng et al., 2013 [74]

*Posttranscriptional *				
ClpXP	EHEC	Regulating intracellular protein levels of GrlR and RpoS	Positively regulates LEE expression	Tomoyasu et al., 2005 [56]; Iyoda and Watanabe, 2005 [37]
CsrA	EPEC	Binding to the leader segment of the *sepL espADB* mRNA and enhancing the steady-state transcript and protein levels	Positively regulates LEE expression	Bhatt et al., 2009 [55]
DegP	EPEC	Protease and chaperone activity	Represses LEE expression	Macritchie et al., 2012 [52]
DsrA	EHEC	Affecting the levels of functional transcripts of *ler *	Activates LEE expression	Laaberki et al., 2006 [58]
Hfq	EHEC	Acting in *ler* and *grlRA* mRNA	Activates or represses LEE expression, depending on the strain background	Shakhnovich et al., 2009 [63]; Hansen and Kaper, 2009 [64]; Kendall et al., 2011 [65]
RNaseE	EHEC	Splicing at the C-terminal end of *sepL* in the precursor *sepL espADB* transcript	Generates the *sepL* and espADB transcripts	Lodato and Kaper, 2009 [53]
RpoS	EHEC	Binding to *ler* prom oter	Activates or represses LEE expression, depending on the strain background	Sperandio et al., 1999 [57]; Laaberki et al., 2006 [58]; Tomoyasu et al., 2005 [56]; Iyoda and Watanabe, 2005 [37]

*Quorum sensing *				
KdpE	EHEC	Binding to *ler *promoter	Activates LEE expression	Njoroge et al., 2013 [70]
QseA	EPEC, EHEC	Binding to *ler *promoter	Activates LEE transcription	Sperandio et al., 2002 [80]; Sircili et al., 2004 [71]
QseC	EHEC	Phosphorylation of KdpE RR	Activates LEE expression	Sperandio et al., 2002 [80]
QseD	EHEC	Binding to *ler* promoter	Represses LEE transcription	Habdas et al., 2010 [83]
QseE	EHEC	Inhibiting RcsB RR transcription	Represses LEE expression	Reading et al., 2007 [81]; Njoroge et al., 2013 [70]
RcsB	EHEC O157:H7; EHEC Sakai		Activates LEE transcription	Njoroge et al., 2013 [70]
GlmY/GLMz	EHEC		Regulates transcription of LEE4 and LEE5	Gruber and Sperandio, 2014 [66]
